# Circ_0067934: a circular RNA with roles in human cancer

**DOI:** 10.1007/s13577-023-00962-y

**Published:** 2023-08-17

**Authors:** Liqing Yu, Jiacheng Zheng, Jiali Yu, Yujun Zhang, Huoli Hu

**Affiliations:** 1 The First Affiliated Hospital of Nanchang University, Nanchang, 330006 Jiangxi Province China; 2 The Second Clinical Medical College of Nanchang University, Nanchang, 330006 Jiangxi Province China; 3 The First Clinical Medical College of Nanchang University, Nanchang, 330006 Jiangxi Province China

**Keywords:** Circ_0067934, CircRNAs, Cancer, Mechanism, Biomarker

## Abstract

A circular RNA (circRNA) is a non-coding RNA (ncRNA) derived from reverse splicing from pre-mRNA and is characterized by the absence of a cap structure at the 5′ end and a poly-adenylated tail at the 3′ end. Owing to the development of RNA sequencing and bioinformatics approaches in recent years, the important clinical value of circRNAs has been increasingly revealed. Circ_0067934 is an RNA molecule of 170 nucleotides located on chromosome 3q26.2. Circ_0067934 is formed via the reverse splicing of exons 15 and 16 in PRKCI (protein kinase C Iota). Recent studies revealed the upregulation or downregulation of circ_0067934 in various tumors. The expression of circ_0067934 was found to be correlated with tumor size, TNM stage, and poor prognosis. Based on experiments with cancer cells, circ_0067934 promotes cancer cell proliferation, migratory activity, and invasion when overexpressed or downregulated. The potential mechanism involves the binding of circ_0067934 to microRNAs (miRNAs; miR-545, miR-1304, miR-1301-3p, miR-1182, miR-7, and miR-1324) to regulate the post-transcriptional expression of genes. Other mechanisms include inhibition of the Wnt/β-catenin and PI3K/AKT signaling pathways. Here, we summarized the biological functions and possible mechanisms of circ_0067934 in different tumors to enable further exploration of its translational applications in clinical diagnosis, therapy, and prognostic assessments.

## Introduction

Owing to the development of ribonucleic acid (RNA) sequencing and bioinformatic approaches in recent years, the Human Genome Project has progressed steadily. To date, more than 20,000 genes have been identified [1, 2]. Further, approximately 95% of RNAs, also known as non-coding RNAs (ncRNAs), are transcribed without post-transcriptional translation [3]. Genes encoding these types of RNAs are usually located in the non-exon part of the genome, with well-established roles in the regulatory processes of DNA-RNA–protein levels. “Housekeeping” ncRNAs, such as transfer (t) RNAs and ribosomal (r)RNAs, are involved in translation [4, 5]. “Regulatory” ncRNAs include long non-coding RNAs (lncRNAs), microRNAs (miRNAs), small interfering RNAs (siRNAs), piwi-interacting RNAs (piRNAs), and circular RNAs (circRNAs) [6]. These ncRNAs are expressed at specific times in response to pathophysiological processes by interfering with messenger RNA (mRNA) expression or binding of other ncRNAs to corresponding proteins [7–9].

circRNAs were first identified by Sanger et al. in 1976. Based on the findings of these researchers, virosomes are cyclic RNA molecules [10]. Since this discovery, circRNAs have also been identified in studies on fungi [11], mice [12, 13], and humans [14]. Unlike other linear RNAs, circRNAs are constructed by reverse splicing of pre-mRNA, which results in the absence of a cap structure at the 5’ ends and poly-adenylated tails at the 3’ ends. Owing to this special construction, circRNAs have a markedly longer half-life due to their stronger resistance to exonuclease degradation. Studies have emphasized that circRNAs are conserved RNAs that are specifically expressed in various cell types and developmental phases [10, 15, 16]. Approximately 343 to more than 1,000 circRNAs have been identified in body fluids, such as exosomes [17], plasma [18, 19], urine, and saliva [20, 21], and can be easily detected using non-invasive biopsies [22].

Multiple cancer-associated circRNAs have been identified to date, which play a role in regulating gene expression and impact various aspects of cellular processes, including cell cycle, proliferation, apoptosis, invasion, and drug resistance [2, 23–26]. Given the multitude of circRNA types, it is challenging to determine the specific mechanisms of each circRNA in different diseases. Additionally, the same circRNA may play distinct roles in disease progression, such as cell apoptosis, autophagy, and ferroptosis [24, 27–29]. Understanding how to maintain a balance between these different types of regulations requires further exploration. Therefore, larger-scale clinical trials and more thorough, in-depth functional studies for each category of circRNA are advised.

Circ_0067934 is an emerging circRNA from protein kinase C Iota (PRKCI) [30]. Owing to a study comprising fifty-one eophageal squamous cell carcinoma (ESCC) samples, Xia et al. first reported increased expression of circ_0067934. Additionally, their analysis revealed abnormal expression associated with the tumor-node-metastasis (TNM) phase and poor survival [31]. Notably, the occurrence and progression of other cancers associated with circ_0067934 have also been determined in recent years. For instance, circ_0067934 has been demonstrated to promote the growth and metastasis of Hepatocellular carcinoma (HCC) by modulating the miR-1324/FZD5/Wnt/β-catenin axis [32]. The hsa_circ_0067934/hsa-mir-4705/BMPR1B axis may be involved in the development of Gastric cancer (GC), according to He et al. [33]. Enhanced expression of circ_0067934 in cervical cancer patients is associated with lymph node metastasis and poor prognosis. And inhibition of circ_0067934-mediated regulation of the epithelial-to-mesenchymal transition process can suppress proliferation, migration, and invasion of Non-small cell lung cancer (NSCLC) cells [34]. Moreover, ferroptosis, a distinct form of programmed cell death from apoptosis and autophagy, plays a critical role in tumorigenesis. Some researchers have proposed the involvement of circRNAs in ferroptosis as a novel approach to cancer therapy [35–37]. Wang et al. demonstrated that circ_0067934 was also involved in cancer cell ferroptosis through the miR-545-3p/SLC7A11 signaling pathway [38]. These previous studies suggest that circ_0067934 is implicated in various tumor diseases, and its specific function and mechanisms in ferroptosis may provide a novel and promising perspective for cancer treatment. However, the research on circ_0067934 in cancer progression is still limited, and further efforts are needed to explore the underlying mechanisms. In this review, we discuss the biogenesis, classification, and function of circular RNAs. In addition, the article highlights the biological functions and molecular mechanisms of circ_0067934 in tumors, as well as the research prospects and biological significance of its involvement in ferroptosis for early cancer diagnosis and precision therapy.

### Biogenesis, distribution, and biological function of CircRNAs

**Fig 1:**
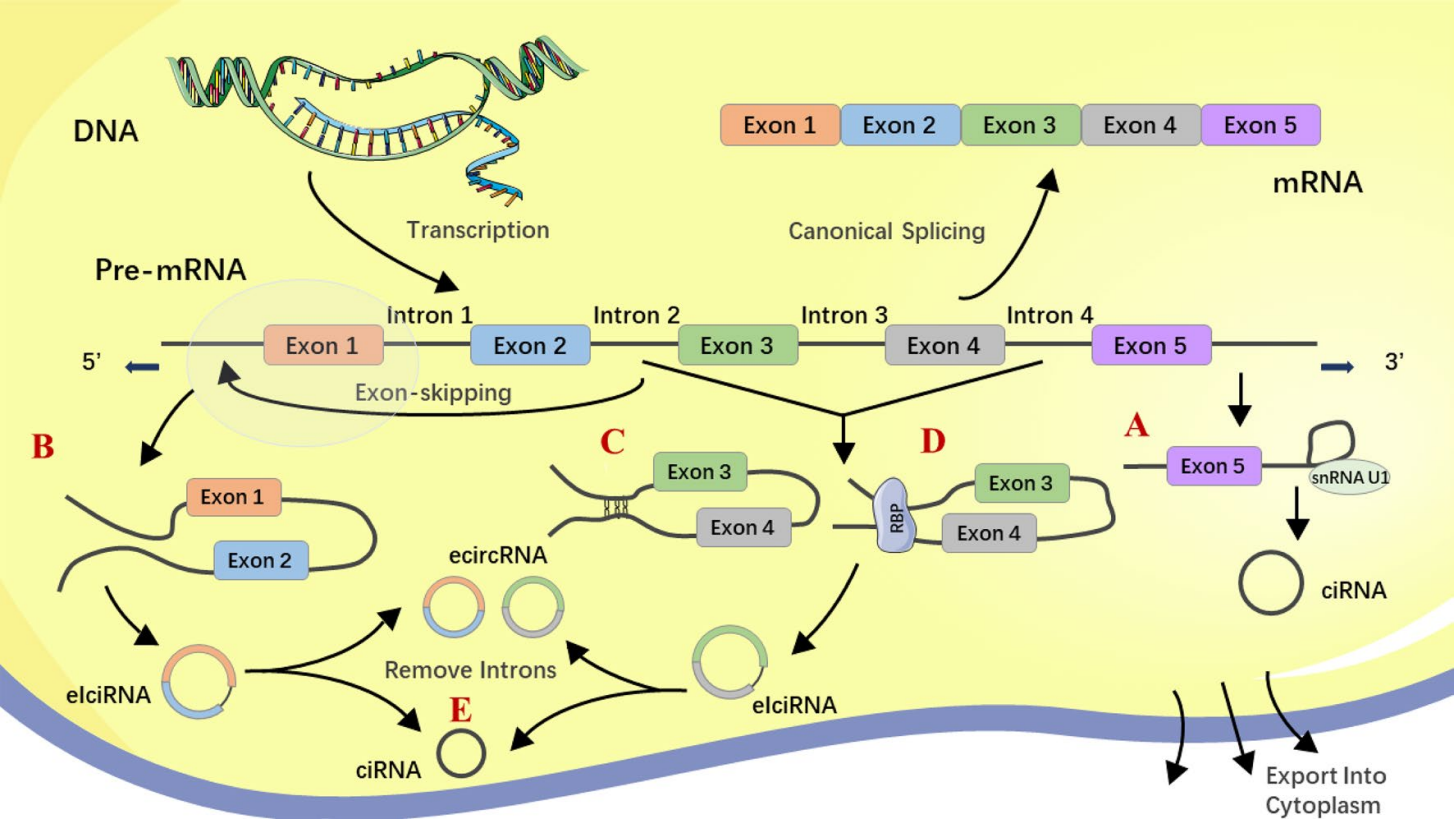
Biogenesis of circRNA. The upper part of the picture shows the canonical transcription and splicing to form mRNA. **A** Circulation of lariat introns. **B** Circularization from exon skipping. **C** Intron pairing-driven circularization. **D** RNA binding protein (RNP)-driven circularization. **E** EcircRNAs after removing introns from eIciRNAs

CircRNAs can be classified into three main types according to their structures: intronic circular RNAs (ciRNAs), exonic circular RNAs (ecircRNAs), and exon–intron circular RNAs (eIciRNAs). Most circRNAs are ecircRNAs, and more than 80% are localized in the cytoplasm. In contrast, ciRNAs and eIciRNAs are mainly localized in the nucleus [39, 40]. Except for circRNAs originating from ribosomal RNA (rRNA), transport RNA (tRNA), and circular DNA tumor virus [41, 42], most circular RNAs in eukaryotic cells is lariat‐circulated or back‐spliced from messenger RNA (mRNA) [43]. The GU-rich sequence at the 5’ end and the C-rich sequence at the other side interact to form a lariat structure with the help of small nucleus RNA (snRNA) U1. Thereafter, the intronic lariat is spliced to generate stable ciRNA (Fig. 1A) [41]. In terms of ecircRNAs, one potential explanation may be the exon skipping mechanism (lariat-driven circularization). Partially folded precursor mRNAs cause two splice acceptor sites to jump close to each other to form a lariat structure. Subsequently, the introns in the lariat structure are removed and the exons are connected by phosphodiester bonds to form the ecircRNA structure (Fig. 1B) [44]. Introns and RNA-binding proteins (RBPs) have also been found to play pivotal roles in circularization-derived ecircRNAs. In intron-driven circularization, reverse splicing is aggravated by complementary sequences in upstream and downstream flanking introns (such as ‘ALU’ elements) (Fig. 1C), leading to a secondary structure that forms an eIciRNA or ecircRNA (intron is removed) [45]. RBPs included quaking-5 (QKI-5) [46], NF90/NF110 [47], heterogeneous nuclear ribonucleoprotein L (HNRNPL) [48], and FUS [49] (Fig. 1D). RBPs promote the cyclization of exons by combining with the flanking introns of transcripts to produce eIciRNAs and ecircRNAs after the removal of introns (Fig. 1E). According to Zhang et al., circRNAs can also be informed by non‐linearly reverse splicing in pre‐mRNA, including one exon loop, or two or more exon loops [50–53].

**Fig 2:**
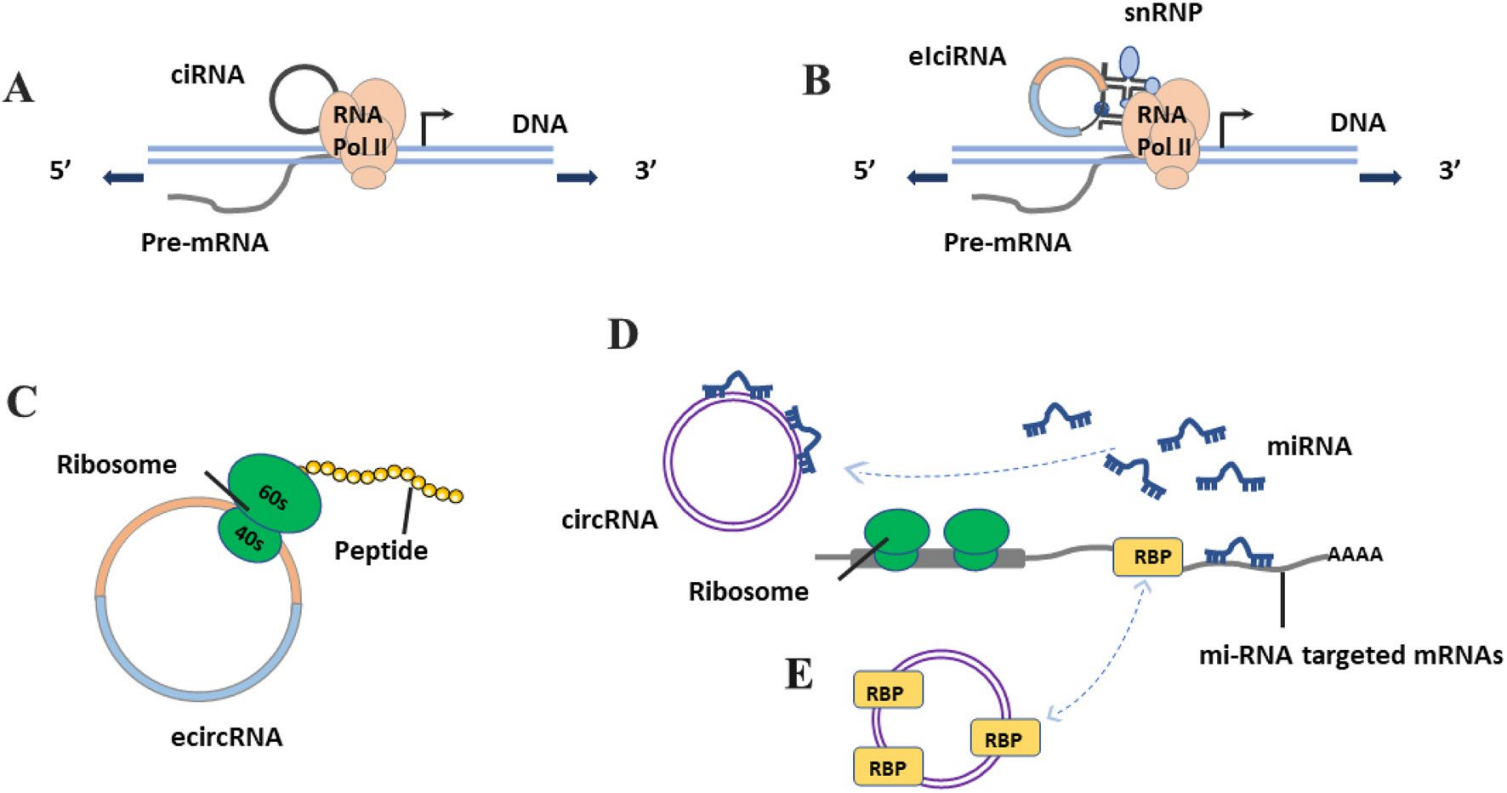
The biological functions of circRNAs.** A** and **B** show the transcriptional regulatory functions of ciRNA and eIciRNA, respectively. **C** Protein-coding potential. **D** Acting as miRNA sponges. **E** Interactions with RBPs

Circular RNAs (circRNAs) are products of variable splicing and have multiple biological functions. Regulating the expression of parental genes is one of the most prominent biological functions and is mainly performed by ciRNAs and eIciRNAs in the cytoplasm. ciRNAs, such as c-sirt7 and ci-ankrd52, interact with RNA polymerase II (Pol II) to modulate positive parental transcription (Fig. 2A) [39, 54]. According to Li et al., eIcircRNAs, such as circPAIP2 and circEIF3J, bind to the small nuclear ribonucleoprotein U1 (snRNP U1) and Pol II to promote host gene transcription (Fig. 2B) [39]. As most circRNAs originate from exons and are located in the cytoplasm, ecircRNAs can be translated. Owing to increasing evidence, 5’ UTR N6‐methyladenosine (m6A)-driven translation is considered the potential translation mechanism of ecircRNAs with ribosome entry site (IRES) and open reading frame (ORF) (Fig. 2C). CircZNF609 is also one of the previously reported circRNAs with an IRES and ORF to generate proteins via this cap-independent mechanism [55]. Other circRNAs with miRNA response elements (MREs) may play the same role as ceRNAs. These circRNAs serve as sponges for miRNAs and prevent their interaction with their corresponding mRNAs, thereby downregulating or upregulating the expression of the targeted genes (Fig. 2D) [56–58]. Similar to this sponge role, circRNAs with binding sites for RBPs may bind to proteins to affect protein function and hence, gene expression (Fig. 2E). For example, extensive attachment of CircPABPN1 (hsa_circ_0031288) to HuR was reported to prevent the engagement of HUR with PABPN1 mRNA, thereby reducing the transcription of PABPN1 [59]. CiRS-7 (a sponge for miR-7) interacts with Argonaute (AGO) proteins in an miR-7-dependent manner, indirectly restraining tumor progression in various cancers [60, 61].

### Characteristics of Circ_0067934

**Fig 3:**
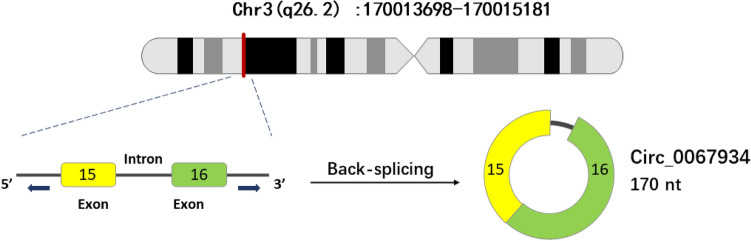
Biogenesis of circ_0067934. Circ_0067934 is generated from chromosome 3q26.2; Exons 15 and 16 are spliced in reverse to shape it; The thick red line indicates its approximate position

Circ_0067934 (OMIM ID: 600,539) is an RNA molecule of 170 nucleotides located on chromosomal region 3q26.2 (Position: chr3: 170,013,698–170015181). Circ_0067934 is formed by the reverse splicing of exons 15 and 16 in the 1483 nucleotides-long gene symbol, PRKCI [31] (Fig. 3). Based on accumulating evidence, circ_0067934 mainly acts as a sponge of microRNAs to further interfere with the regulation of the post-transcriptional processes of the target gene. For example, circ_0067934 promotes tumor progression in GC by sponging miR13013p and regulating KIF23 expression [62]. Liu et al. revealed that circ_0067934 increases Myc expression by suppressing miR-1304 expression, thereby accelerating the proliferation, migration, and invasion of Bladder cancer (BC) [63]. In addition, Circ_0067934 may contribute to the development of HCC through the induction of miR-1324/FZD5 /Wnt/β-catenin signaling [32].  > hsa_circ_0067934|ENST00000295797 GTTATTTTGGAAAAACAAATTCGCATACCACGTTCTCTGTCTGTAAAAGCTGCAAGTGTTCTGAAGAGTTTTCTTAATAAGGACCCTAAGGAACGATTGGGTTGTCATCCTCAAACAGGATTTGCTGATATTCAGGGACACCCGTTCTTCCGAAATGTTGATTGGGATATG (http://bis.zju.edu.cn/CircFunBase/detail.php?name=hsa_circ_0067934).

### Circ_0067934 in human cancers

**Fig 4:**
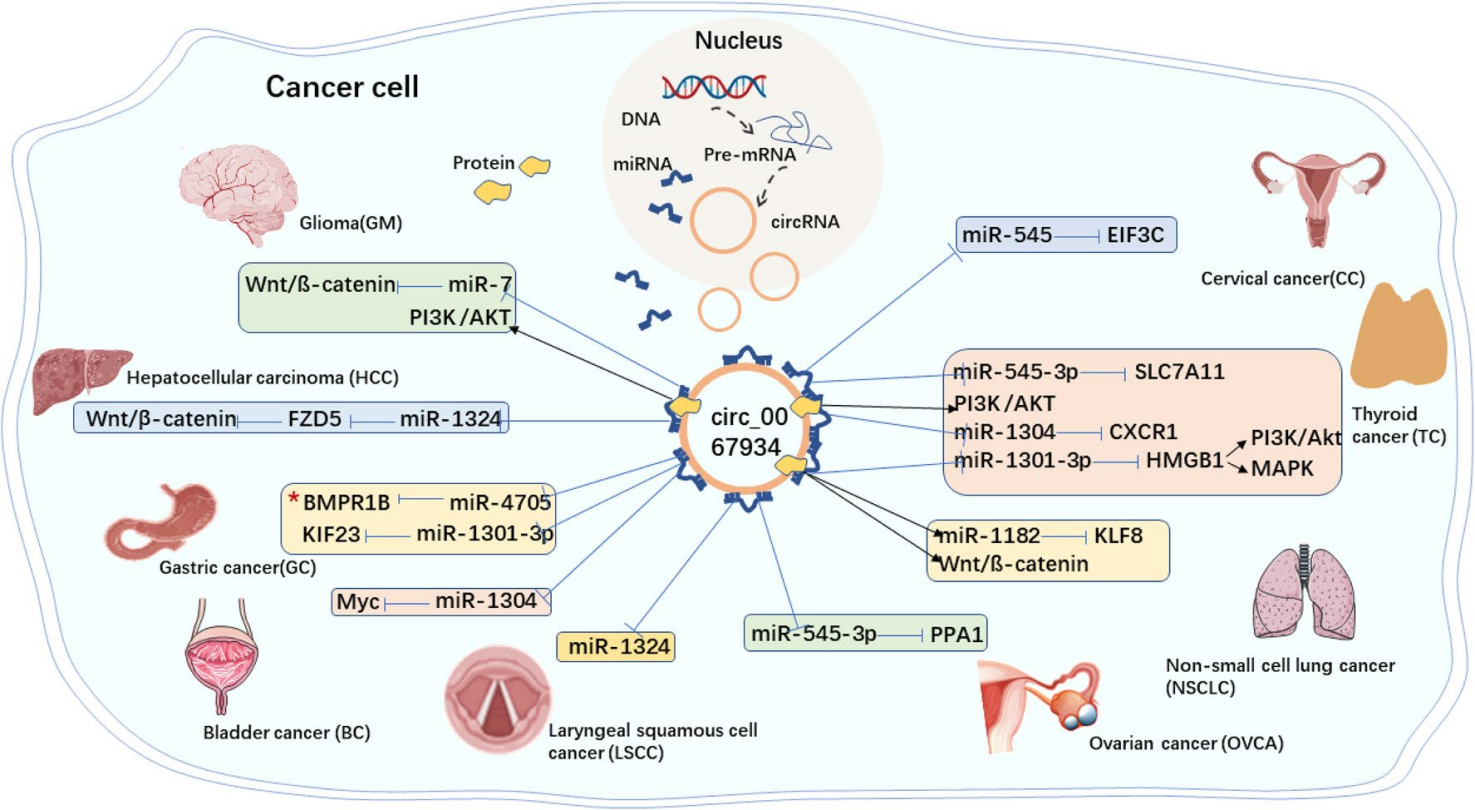
Molecular mechanism of circ_0067934 in human cancers**.** Standard-shaped black arrow represents stimulation; T-shaped blue arrow represents inhibition. Circ_0067934 promotes cancer development in pathways marked with red asterisks “circ 0067934/miR-4705/BMPR1B” and inhibits cancer development in other pathways

As a carcinogen, circ_0067934 is abnormally expressed in various human malignant tumors and participates in post-transcriptional regulation. Circ_0067934 has been established to be closely associated with tumor proliferation, migration, and invasion. The potential molecular mechanisms are listed in Table 1 and Fig. 4.

**Table 1:** Functional characterization of circ_0067934 in human cancers

Cancer types	Dysregulation	Function role	Signaling pathways	References
Cervical cancer (CC)	Upregulation	Promote proliferation, colony formation, migration, invasion, and EMT	miR-545/EIF3C	[65]
Thyroid cancer (TC)	Upregulation	Attenuate ferroptosis of thyroid cancer cells	miR-545-3p/SLC7A11	[38]
Thyroid cancer (TC)	Upregulation	Promote cell proliferation, migration, and invasion; inhibit apoptosis	PI3K/AKT	[71]
Thyroid cancer (TC)	Upregulation	Trigger apoptosis in vitro; curb proliferation, migration, and invasion of cells in vitro; repress tumor growth in vivo	miR-1304/CXCR1	[30]
Papillary thyroid carcinoma (PTC)	Upregulation	Promote growth, colony formation, migration, invasion, EMT, and tumor xenograft growth	miR-1301-3p/HMGB1 PI3K/Akt MAPK	[70]
Non-small cell lung cancer (NSCLC)	Upregulation	Promote proliferation, migration, invasion, and EMT; inhibite apoptosis	miR-1182/KLF8 Wnt/β-catenin	[78]
Glioma (GM)	Upregulation	Promote cell proliferation, migration and invasion	miR-7/ Wnt/β-catenin	[81]
Glioblastoma (GBM)	Upregulation	Promote proliferation, metastasis and EMT; inhibite apoptosis	PI3K/AKT	[82]
Hepatocellular carcinoma (HCC)	Upregulation	Promote proliferation, migration, invasion; reduce apoptosis	miR-1324/FZD5/WNT/β-catenin	[32]
Gastric cancer (GC)	Downregulation	Suppress proliferation, differentiation and apoptosis	miR-4705/BMPR1B	[33]
Gastric cancer (GC)	Upregulation	Promote proliferation, migration and invasion	miR-1301-3p/KIF23	[62]
Bladder cancer (BC)	Upregulation	Promote proliferation, migration and invasion	miR-1304/Myc	[63]
Laryngeal squamous cell cancer (LSCC)	Upregulation	Promote proliferation, migration	miR-1324	[69]
Ovarian cancer (OVCA)	Upregulation	Promote proliferation, invasion and cisplatin (DDP) resistance; reduce phosphorylation of the pro-apoptotic JNK signaling	miR-545-3p/PPA1	[109]
Esophageal squamous cell carcinoma (ESCC)	Upregulation	Promote proliferation and migration	NA	[31]

Recombinant solute carrier family 7, Member 11 = SLC7A11. Epithelial-to-mesenchymal transition = EMT. Phosphatidylinositol 3 kinase = PI3K. Protein kinase B = AKT. *CXC* chemokine receptor 1 = CXCR1. Mitogen-activated protein kinase = MAPK. High mobility group protein 1 = HMGB1. Kruppel-like factor 8 = KLF8. Kinesin family member 23 = KIF23. Frizzled class receptor 5 = FDZ5. Bone morphogenetic protein receptor type 1B = BMPR1B. Pyrophosphatase 1 = PPA1. Eukaryotic translation initiation factor 3 subunit C = EIF3C

#### Cervical cancer (CC)

CC is the second most common cause of malignant tumors in the female reproductive system, accounting for approximately 18.4% of all female malignant tumor deaths [32]. Some geographic disparities have been found for CC. Moreover, CC is found to occur at an early age, posing a grave threat to the well-being of women [64]. As multiple genes and various pathological processes are involved in CC, its causes and progression are complicated. Hu et al. found that circ_0067934 facilitates CC progression by acting as a sponge for miR-545 [65]. Silencing of circ_0067934 promoted miR-545 expression but inhibited EIF3C expression. CC progression can be slowed down by increasing miR-545 levels and decreasing EIF3C levels.

#### Thyroid cancer (TC)

As the most prevalent endocrine tumor, the incidence of TC has been increasing rapidly in recent years, affecting more women than men. Papillary thyroid carcinoma and follicular thyroid carcinoma remain as the most common histological types of TC. These types are referred to as differentiated TC and have significant variability [66, 67]. Although differentiated TC has a favorable prognosis, distant metastasis still occurs in approximately 7–23% of TC patients, serving as the main cause of TC-related death [68]. Based on accumulating research, circ_0067934, a powerful tumor promoter, sponges different miRNAs. Wang et al. discovered that circ-0067934 is overexpressed in TC and promotes proliferation, migratory movement, and invasion via the miR-545-3p/SLC7A11 signaling pathway [38]. In contrast to the findings of Zhang, Dong et al. propose that circ0067934 has the potential to act as a molecular sponge for multiple miRNAs, such as hsa-mir-4705 in GC [33] and miR-1324 in Laryngeal squamous cell cancer (LSCC) [69]. In addition to TC tumor progression, circ_0067934 expression was increased by the inhibition of miR-1301-3p and activation of HMGB1, PI3K/Akt, and MAPK. Knockdown of circ_0067934 represses tumor xenograft proliferation [70]. However, these studies have primarily elucidated the crucial role of Circ_0067934 in the progression of thyroid cancer using cell and mouse models, limiting their direct relevance to human subjects. Further research is warranted to explore the clinical significance of Circ_0067934. In an experimental study involving 50 tissue samples from TC patients, Zhang et al. demonstrated that the knockdown of circ_0067934 could downregulate CXCR1 expression through miR-1304 sponging, thereby expediting apoptosis and inhibiting proliferation, migratory movement, and TC cell invasion [30]. Another study conducted by Wang et al. utilized quantitative Real Time-Polymerase Chain Reaction (qRT-PCR) to measure the expression of circ_0067934, which was significantly upregulated in cancer cells [71]. Knockdown of circ_0067934 inhibited the EMT and PI3K/AKT signaling pathways, ultimately inhibiting cell proliferation, migratory activity, invasion, and apoptosis. However, due to limitations in their hospital setting, the number of patients included in the study was relatively small. Additionally, downstream pathways of the EMT and PI3K/AKT signaling pathways have not been investigated. Therefore, further research involving a larger cohort of patients is necessary to address these limitations and provide a more comprehensive understanding of the topic.

#### Non-small cell lung cancer (NSCLC)

According to a survey conducted in more than 100 countries, lung cancer is associated with the highest incidence and mortality rates among all malignant tumors [72]. There are two major types of lung cancer: small cell lung cancer and non-small cell lung cancer (NSCLC), with NSCLC accounting for 80–85% of cases and is mainly composed of adenocarcinoma and squamous cell carcinoma [73]. G Chassagnon et al. proposed a new TNM classification for NSCLC, which could be a key part of the initial evaluation [74] and of crucial clinical significance for early diagnosis and treatment [75]. Wen-Juan Liu et al. demonstrated that compared with traditional methods, targeted therapy could significantly improve the health status and overall survival of patients with NSCLC [76]. Within NSCLC tissues, Zou et al. and Wang et al. found the overexpression of circ_0067934. These researchers also found that overexpression was significantly associated with poor overall survival (OS) [34, 77]. Zou et al. knocked down circ_0067934 and observed significant inhibition of tumor proliferation [77]. Zhao et al. discovered that knocking down circ_0067934 not only increased miR-1182 expression in NSCLC tissues but also inhibited KLF8 expression [78]. The upregulation of circ_0067934 and KLF8 or the downregulation of miR-1182 facilitated NSCLC progression.

#### Glioma (GM)

GM is the most common malignant tumor of the central nervous system and typically occurs between 30 and 40 years of age. The incidence of GM is greater in men than in women and has been increasing over the past few years. Glioblastoma represents the most malignant subtype of glioma, with the largest proportion showing a significantly increasing trend in the population after 54 years old [79]. Patients with glioblastomas have lower survival rates than those with other types of GM [80]. Pei et al. identified circ_0067934 as an overexpressed gene in GM [81]. Circ_0067934 binds with miR-7, regulating the Wnt/β-catenin axis and promoting cancer cell proliferation, invasion, and migratory movement. Xin et al. provided evidence of the carcinogenic role of circ_0067934, which upregulated the PI3K/AKT pathway to promote cancer cell proliferation, invasion, and metastasis [82].

#### Hepatocellular carcinoma (HCC)

In 2018, 841,000 new cases of liver cancer were reported, making it the sixth most frequent disease globally and the fourth most common cause of cancer-related deaths [83, 84]. HCC accounts for 75–85% of new cancer cases. Early stage liver cancer can be treated by surgical resection, transplantation, or ablation. However, liver cancer is difficult to diagnose in many patients, leading to a poor prognosis [85]. Therefore, new biomarkers are critical for detecting the disease at the earliest possible phase. Previous studies have shown that circMTO1, circRNP, and circVAMP3 inhibit the progression of HCC [86–88]. However, many circRNAs remain unknown. Zhou et al. revealed that circ_0067934 levels were significantly higher in HCC tissues than in non-tumor tissues. Moreover, a positive correlation was found between circ_0067934 expression and tumor size, TNM phase, and microvascular invasion [89]. Similar results were obtained in another study. Circ_0067934 expression was discovered to be significantly higher in HCC tissues than in adjacent normal tissues, and its knockdown was found to significantly reduce proliferation, movement, and invasiveness [32]. Such finding may be due to the upregulation of miR-1324, which targets the 3’ non-coding region of FZD5mRNA and suppresses the FZD5, nuclear β-catenin, and cyclin D1 expression within HCC cells, after the downregulation of circ_0067934. Circ_0067934 was found to modulate the miR-1324/FZD5/Wnt/-catenin axis, which has significant clinical advantages in the investigation of therapeutic targets for HCC intervention [32].

#### Gastric cancer (GC)

In 2018, GC resulted in 1,033,701 new cases and 782,685 fatalities [90]. Patients with GC are usually symptom-free, and as no effective methods are available to detect GC, the rate of early diagnosis is very low. Therefore, > 70% of patients are diagnosed with advanced GC [91]. At present, surgery is the only treatment option for GC; however, patients with phase III GC have a 5-year survival rate of less than 50%. As a result, new treatment strategies are needed. In recent studies, circ_0067934 was found to be involved in the progression of GC and may become a new prognostic or diagnostic marker and a therapeutic target. According to He et al., BMPR1B expression was downregulated in GC when miR4705, which is downstream of circ_0067934, was downregulated [28]. This finding may be related to the hindrance of the Wnt signaling pathway, calcium regulatory pathway, and the binding of transforming growth factor-β (TGF-β) & activator protein. By regulating both the Hippo and signaling pathways that regulate stem cell pluripotency, the circ_0067934/has-mir-4705/BMPR1B axis promotes the occurrence and development of GC. However, Xu et al. reported the upregulation of circ_0067934 in GC cells [62]. Circ_0067934 was found to target miR-1301-3p, and the overexpression of miR-1301-3p, which targets KIF23, prevented the proliferation of GC cells. The effects of circ_0067934 silencing and miR-1301-3p overexpression on cells were reversed by the overexpression of KIF23. Therefore, circ_0067934 may regulate GC cell proliferation, invasion, and migration through the miR-1301-3p/KIF23 signaling axis [62].

#### Esophageal squamous cell carcinoma (ESCC)

A total of 604,100 new cases of esophageal cancer and 544,100 deaths were reported in 2020, with ESCC accounting for most of these cases [92]. Due to the lack of early specific symptoms, many patients with ESCC are diagnosed with advanced or distant metastases, resulting in a low 5-year survival rate and a tendency to relapse after surgery. Notably, studies on neoadjuvant chemoradiotherapy followed by minimally invasive esophagectomy, immunotherapy combined with cytotoxic drugs, and biomarkers have made significant progress [93–95]. Using esophageal cancer cell lines as a model to evaluate the expression of circ _0067934, Xia et al. found that the inhibition of circ_0067934 significantly reduced the invasion ability of cancer cells compared to control cells [31]. Therefore, these researchers concluded that circ_0067934 contributes to cancer cell motility and migration, which may affect cell cycle status and become a new therapeutic target for ESCC. Elevated circ_0067934 expression was also reported to be associated with decreased differentiation, I-II T stage, and I-II tumor node metastasis (TNM) stage compared with normal expression in adjacent tissues [31]. Overall, circ_0067934 expression is increased in ESCC tumor tissues, and its downregulation inhibits the proliferation and migration of ESCC cells.

#### Bladder cancer (BC)

Over 500,000 new infections and approximately 200,000 fatalities are estimated to occur worldwide annually due to BC. In addition to traditional surgical resection, radiotherapy, and chemotherapy, immunotherapy is another potential approach for the treatment of BC [96]. Nonetheless, survival after five years for advanced BC remains low owing to postoperative recurrence and distant metastatic disease [97]. Therefore, potential therapeutic targets and biomarkers of BC must be identified. Studies have shown that circRNAs, such as circSLC8A1 [98], circRNA-Cdr1as [99], and circular RNA BCRC-3 [100], contribute to the occurrence, development, and metastasis of BC. Recently, circ_0067934 was confirmed as a potential diagnostic marker for patients with BC. Liu et al. found a significant reduction in the migrating and invasive ability of T24 cells in the circ_0067934 group, including a reduction in their ability to proliferate compared to those of cells in the siNC group [63]. This finding may be related to the role of circ_0067934 as a sponge for miR-1304, thereby upregulating Myc expression to promote BC proliferation, migratory activity, and invasion. In addition, their study revealed that patients with circ_0067934 expression were significantly less likely to survive for five years and have disease-free survival, and the expression of circ_0067934 was significantly associated with tumor size, advanced phase, and metastasis to lymph nodes[63].

#### Laryngeal squamous cell carcinoma (LSCC)

In 2018, 890,000 newly diagnosed cases and 450,000 fatalities owing to head and neck squamous cell carcinoma were recorded worldwide, of which approximately 20% were caused by LSCC [101, 102]. Laryngeal cancer, which is diagnosed at an early stage, is generally treated surgically with satisfactory outcomes. Radical laryngectomy (also known as total or near-total laryngectomy) may be performed in patients with advanced LSCC, followed by adjuvant treatment or systemic chemoradiotherapy [103]. The 5-year overall survival rate for locally advanced laryngeal cell carcinoma remains below 50%, with potential postoperative recurrence [104]. Therefore, potential therapeutic targets and biomarkers of LSCC have great clinical value [105]. Recent research revealed that high circ_0067934 expression is closely associated with tumor size, the presence of lymph node metastases, and poor prognosis in LSCC. After transfecting circ_0067934 into the TU212 and TU686 cell lines, Chu et al. determined the effect of circ_0067934 on cell proliferation. After circ_0067934 knockdown, the survival rates of TU212 and TU686 cells decreased, and the number of colonies formed by laryngeal carcinoma cells decreased [69]. The migrating cells were also significantly reduced when circ_0067934 expression was inhibited in LSCC cells. Through sponging miR-1324, circ_0067934 may promote laryngeal cancer cell proliferation and migratory activity.

#### Ovarian cancer (OVCA).

A total of 239,000 reported cases of OVCA and 152,000 fatalities due to this disease have been recorded annually. A debulking procedure is combined with maintenance chemotherapy comprising platinum and taxane to serve as the current standard of care. However, OVCA is associated with a high recurrence rate after front-line treatment [106, 107]. Currently, most clinical trials involve targeted approaches, including recent efforts to introduce immunotherapy into the field of OVCA treatment [108]. Notably, the role of circRNAs in OVCA has been proposed. By assessing the expression of circ_0067934 in OVCA cells and the medical characteristics of patients, Yin et al. discovered that circ_0067934 was strongly expressed in OVCA samples and was associated with an advanced tumor phase and metastasis to lymph nodes [109]. Their studies revealed that circ_0067934, which acts as a sponge for miR-545-3p, upregulated the translational expression of PPA1, thereby enhancing the proliferation, invasion, and anti-DDP abilities of the anti-DDP A2780/DDP cell line, and suppressing the pro-apoptotic signaling pathway.

### Ferroptosis related to Circ_0067934

The imbalance between abnormal proliferation and apoptosis in cancer cells is a significant biological characteristic of malignant tumors. In 2012, Dixon et al. proposed a novel form of regulated cell death called ferroptosis [110]. Ferroptosis is characterized by iron-dependent accumulation of lipid peroxides, resulting in cellular damage [36, 111]. Specifically, Fe3 + enters the cell and is reduced to Fe2 + by the action of STEAP3. It is then released into the cytoplasm via the divalent metal transporter 1 (DMT1), leading to ROS accumulation and subsequent induction of lipid peroxidation and ferroptosis [112, 113]. The glutathione pathway plays a crucial role in ferroptosis. For instance, circACAP2 promotes malignant progression in CC by competitively binding to the key ferroptotic protein GPX4 [114]. In recent years, the role of ferroptosis has gradually been revealed in various diseases [36, 115–117]. Multiple clinical studies, supported by extensive research and validation using large sample sizes, have demonstrated that drugs can exert anti-tumor effects by inducing ferroptosis [118–120]. For instance, in the treatment of breast cancer, the use of metformin inhibits SLC7A11-induced ferroptosis and suppress tumor growth. Curcumin, on the other hand, inhibits tumor progression by activating autophagy-induced ferroptosis [37]. Additionally, certain cytokines can also induce ferroptosis, such as disulfiram/copper in nasopharyngeal cancer [121]. Hence, the induction of ferroptosis may represent a promising strategy for effectively inhibiting tumor metastasis.

It is worth noting that besides regulating cellular autophagy and apoptosis to impact cancer progression, recent research has revealed the involvement of ferroptosis in the modulation of cancer progression by circRNAs. The interaction of cIARS with the RBPs ALKBH5 has been shown to inhibit its ability to promote ferroptosis [122]. Additionally, circBCAR3 has been reported to promote ferroptosis by enhancing the expression level of the transporter protein-1 through its association with miR-27a-3p [123]. Furthermore, circ0007142 [124], circGFRA1 [125], circABCB10 [126] and CircLMO1 [127] can regulate the expression of ferroptosis-related genes through miRNA-mediated mechanisms. Collectively, these findings suggest that ferroptosis involving circRNAs may emerge as a novel approach for cancer therapy. Regarding the specific circRNA circ_0067934, which has been implicated in ferroptosis, Wang et al. [128]. demonstrated that simultaneous inhibition of miR-545-3p and SLC7A11 overexpression can suppress ferroptosis caused by circ_0067934 knockout in TC. The competitive binding of circ_0067934 with miR-545-3p upregulates SLC7A11 expression, inhibiting ferroptosis and promoting the development of TC. However, as research regarding the involvement of circ_0067934 in ferroptosis in cancer development remains relatively limited, further investigation is necessary to elucidate the mechanistic role of circ_0067934 in regulating ferroptosis in other types of cancer.

### Conclusion and future perspectives

Circ_0067934 is a cyclic non-coding RNAs that has been reported to be significantly associated with tumor occurrence and development over the past few years. The present review focused on the differential expression, biological functions, and molecular mechanisms of circ_0067934 in various diseases. Dual-luciferase reporter gene assay, colony formation, fluorescence in situ hybridization and qRT-PCR are commonly used methods in circRNA research. Except for the findings of He et al., circ_0067934 expression was found to be higher in cancer tissues than in normal tissues [28]. Of note, Xin et al. [82]'s study did not conduct COX regression analysis, and both Xia et al. [31] and Liu et al. [63]'s studies had a sample size of only 50 participants. Further research is needed to address these limitations.

Epigenetic changes, such as gene mutations, may play a role in circRNA dysregulation in diseases. A significant correlation was found between abnormal expression and tumor size, TNM phase, and survival rate. According to the experiments of cancer cells, the overexpression or underexpression of circ_0067934 resulted in increased EMT, proliferation, migratory activity, and invasion of tumor cells. In terms of its primary mechanism, circ_0067934 functions as a sponge for miRNAs to regulate the post-transcriptional expression of genes. In this context, circ_0067934 may be useful at identifying novel targets and biomarkers for diagnostic use in tumor treatment.

The competing endogenous RNA (ceRNA) regulation mechanism was first described in 2011 by Salmena et al. [129]. According to the hypothesis, miRNAs and non-coding RNA limit transcript expression by binding to MREs [130]. Coding genes and non-coding genes interact with each other to regulate their expression levels through the competitive binding of miRNAs. Circ_0067934 promotes tumor progression by competitively binding to various miRNAs (miR-545 [38, 65, 109], miR-1304 [30, 63], miR-1301-3p [70], miR-1182 [78], miR-7 [81], miR-1324 [32, 69]); other mechanisms include inhibiting the Wnt/β –catenin [78] and PI3K/AKT [71, 82] signaling pathways. A study involving 51 patients with ESCC also found that circ_0067934 is upregulated in tumor tissues; however, the underlying mechanisms remain unknown [31]. Although the role of circ_0067934 in various tumors is understood to some extent, the mechanisms of metabolic reprogramming between circ_0067934 and tumor microenvironment, the regulation of tumor cell stemness, and drug resistance must be urgently elucidated. Several problems must also be resolved before its clinical application. First, circ_0067934 may be abnormally expressed in other cancers; however, its high specificity and low toxicity in cancer diagnosis have not been clarified. Second, the potential of circ_0067934 as a biomarker must be verified in multicenter cohort studies with larger sample sizes. Notably, circRNAs are enriched in extracellular vesicles and are stably present in the blood, enabling the specific targeting of molecular drugs. However, the role of circ_0067934 in exosomes remains unclear.

Collectively, increasing research has provided novel insights into the biological characteristics of circ_0067934. However, these studies remain at the cellular and animal levels. Future studies should focus on translational applications in clinical diagnosis, prognostic assessments, and potential treatment targets.

## Data Availability

Data availability is not applicable to this article as no new data were created or analyzed in this study.
